# Heart Failure with Preserved Ejection Fraction: How to Deal with This Chameleon

**DOI:** 10.3390/jcm13051375

**Published:** 2024-02-28

**Authors:** Fabiana Lucà, Fabrizio Oliva, Maurizio Giuseppe Abrignani, Stefania Angela Di Fusco, Mauro Gori, Simona Giubilato, Roberto Ceravolo, Pier Luigi Temporelli, Stefano Cornara, Carmelo Massimiliano Rao, Giorgio Caretta, Andrea Pozzi, Giulio Binaghi, Alessandro Maloberti, Concetta Di Nora, Irene Di Matteo, Anna Pilleri, Sandro Gelsomino, Carmine Riccio, Massimo Grimaldi, Furio Colivicchi, Michele Massimo Gulizia

**Affiliations:** 1 Cardiology Department, Grande Ospedale Metropolitano, 89129 Reggio Calabria, Italy; 2 Cardiology Department De Gasperis Cardio Center, Niguarda Hospital, 20162 Milan, Italyalessandro.maloberti@ospedaleniguarda.it (A.M.);; 3 Operative Unit of Cardiology, P. Borsellino Hospital, 91025 Marsala, Italy; 4 Cardiology Department, San Filippo Neri Hospital, ASL Roma 1, 00135 Rome, Italy; 5 Cardiovascular Department, Azienda Ospedaliera Papa Giovanni XXIII Hospital, 24127 Bergamo, Italy; 6 Cardiology Department, Ospedale Lamezia Terme, 88046 Catanzaro, Italy; 7 Cardiac Rehabilitation Unitof Maugeri, IRCCS, 28010 Gattico-Veruno, Italy; 8 Cardiology Department, Cannizzaro Hospital, 95126 Catania, Italy; 9 Arrhytmia Unit, Division of Cardiology, Ospedale San Paolo, Azienda Sanitaria Locale 2, 17100 Savona, Italy; stefano.cornara@gmail.com; 10 Levante Ligure Sant’Andrea Hospital, ASL 5 Liguria, 19121 La Spezia, Italy; 11 Cardiology Division, Valduce Hospital, 22100 Como, Italy; 12 Department of Cardiology, Azienda Ospedaliera Brotzu, 09134 Cagliari, Italy; 13 Department of Cardiothoracic Science, Azienda Sanitaria UniversitariaIntegrata di Udine, 33100 Udine, Italy; 14 Cardiovascular Research Institute, Maastricht University, 6229 HX Maastricht, The Netherlands; 15 Cardiovascular Department, Sant’Anna e San Sebastiano Hospital, 81100 Caserta, Italy; 16 Department of Cardiology, General Regional Hospital “F. Miulli”, 70021 Bari, Italy; 17 Cardiology Department, Garibaldi Nesima Hospital, 95122 Catania, Italy; michele.gulizia60@gmail.com

**Keywords:** heart failure with preserved ejection fraction (HFpEF), mineralocorticoid receptor antagonists (MRAs), sodium-glucose cotransporter-2 (SGLT2) inhibitors sacubitril/valsartan chronic

## Abstract

Heart failure with preserved ejection fraction (HFpEF) is characterized by a notable heterogeneity in both phenotypic and pathophysiological features, with a growing incidence due to the increase in median age and comorbidities such as obesity, arterial hypertension, and cardiometabolic disease. In recent decades, the development of new pharmacological and non-pharmacological options has significantly impacted outcomes, improving clinical status and reducing mortality. Moreover, a more personalized and accurate therapeutic management has been demonstrated to enhance the quality of life, diminish hospitalizations, and improve overall survival. Therefore, assessing the peculiarities of patients with HFpEF is crucial in order to obtain a better understanding of this disorder. Importantly, comorbidities have been shown to influence symptoms and prognosis, and, consequently, they should be carefully addressed. In this sense, it is mandatory to join forces with a multidisciplinary team in order to achieve high-quality care. However, HFpEF remains largely under-recognized and under-treated in clinical practice, and the diagnostic and therapeutic management of these patients remains challenging. The aim of this paper is to articulate a pragmatic approach for patients with HFpEF focusing on the etiology, diagnosis, and treatment of HFpEF.

## 1. Introduction

Heart failure (HF) represents a complex disorder consisting of symptoms and signs due to underlying cardiac disorders that result in structural and functional abnormalities.

Making the diagnosis is mandatory, as well as identifying the etiology, in order to choose the most appropriate clinical and therapeutic approach [1].

Although HF represents a uniform clinical conundrum, it has been conventionally divided into three phenotypes according to LVEF value.

In this sense, it has been indicated as HF with reduced ejection fraction (HFrEF), HF with mildly reduced ejection fraction (HFmrEF), and HF with preserved ejection fraction (HFpEF), conditions with LVEF ≤40%, between 41% and 49%, and ≥50%, respectively [2].

Characterized by multiorgan involvement, HFpEF is a leading cause of morbidity, mortality, and hospitalization, with an increasing incidence [3].

Importantly, a greater prevalence of HFpEF in women in comparison to men has been reported [3]. Due to the different phenotypes and complex pathophysiologic mechanisms, HFpEF may be considered a multifactorial and multisystemic syndrome representing a complex issue for clinicians [4]. Indeed, it is mandatory to take into consideration the influence of concurrent comorbidities that exacerbate symptoms influencing prognosis. Moreover, pharmacological and non-pharmacological interventions should be employed in order to enhance the quality of life (QoL) and overall survival of patients with these conditions, diminishing HF hospitalizations (HHF) [4].

While the effectiveness of treatments has been well assessed in HFrEF, more is needed about the benefits of therapies for HFpEF. Sodium/glucose cotransporter-2 inhibitors (SGLT2i) have been recently shown to reduce the risk of cardiovascular (CV) events, particularly HHF [3].

Moreover, the guidelines for HFpEF are mostly focused on prevalent comorbidities, whereas HFpEF management still needs to be clarified [2].

A comprehensive management of HFpEF, including a targeted approach, has also been proposed [4]. Moreover, a multidisciplinary approach is essential for treating these patients with high-quality care.

However, HFpEF undoubtedly represents a diagnostic and therapeutic challenge.

We performed a narrative review involving studies focused on HFpEF, focusing on the pathophysiology, diagnosis, classification, and therapeutic management of patients with HFpEF.

### 1.1. Etiology

The pathophysiological mechanisms involved in HFpEF development remain partially unclear.

Microvascular dysfunction, oxidative stress, inflammation, and senescence have been proposed as etiological factors of HFpEF [5].

Senescence has been shown to be linked to endothelial dysfunction and fibrosis, inducing systemic cardiac, structural, and functional changes [6,7].

A rapid failure of cardiomyocyte functionality occurs in the presence of mitochondrial alterations, increased activity of reactive oxygen species (ROS), and insufficient detoxification and alterations in the extracellular matrix (ECM) structure. Alterations in growth signaling pathways, including insulin-like growth factor-1 (IGF-1), have also been reported in the absence of CVD, confirming the direct relationship between senescence and HFpEF [8]. Circulating molecules such as the vascular endothelial growth factor, IL-1, IL-8 cytokines, matrix metalloproteinase, and the plasminogen activator inhibitor (PAI)-1 have been recognized as being involved in the mechanisms sustaining inflammation and oxidative stress tissue remodeling [5]. This secretory pattern has been hypothesized to promote the development of HFpEF [5,9]. Indeed, inflammation has been proposed to be a leading etiological factor of HFpEF [9]. In obesity, diabetes, and hypertension, systemic inflammation determines detrimental effects on cardiomyocyte metabolism and microvascular function. Tumor necrosis factor (TNF)-alpha and transforming growth factor-beta 1 (TGF-beta1) have been proposed to play a role in atherosclerosis subendocardial ischemia [9] and in HFpEF development [9]. Decreased nitric oxide synthase 3 (NOS3) and endothelialNOsynthase (eNOS) activity have also been described in HFpEF [9].

### 1.2. Age

Age has been shown to play an essential role in HFpEF development, as confirmed by its higher incidence in patients aged over 65 years [10], due to both direct mechanisms related to senescence and increasing comorbidities. Notably, different age-related profiles have been described in patients with HFpEF. Younger patients are more likely to be obese non-white men, differently from the elderly, who are more frequently women with a greater burden of comorbidities [11]. Furthermore, young patients have been reported to have a worse quality of life and a higher likelihood of dying from CV causes, especially sudden death. In contrast, in older people, the mortality rate is higher and more commonly caused by non-CV disorders. Indeed, in the elderly, a high prevalence of multimorbidity and geriatric syndromes has been recognized, including frailty, malnutrition, cognitive impairment, and depressive disorders. In this sense, a geriatric assessment involving several domains is essential for performing a comprehensive and multiparametric evaluation, including the presence of comorbidities, polytherapy, nutritional status, physical and cognitive impairment, depressive symptoms, frailty, falls, and lack of social relationships [11]. Indeed, these factors have been recognized to be highly prevalent in HFpEF, influencing quality of life, hospitalization, and mortality. Physicians can use several tools in routine practice to detect this typical impairment and consequently improve evidence-based strategies. The targeted geriatric assessment (TaGA), investigating various geriatric aspects, including social support, recent hospitalizations, falls, polypharmacy, functional capacity, cognitive status, nutritional well-being, self-rated health, and the presence of depression, demonstrates the capability to evaluate various geriatric domains rapidly, proving prognostic insights in the context of HFpEF [12].

## 2. Medical History and Clinical Examination

Patients with HFpEF require a comprehensive evaluation consisting of more than echocardiographic imaging in order to be correctly identified [1]. Numerous scores and algorithms have been suggested to aid healthcare professionals in characterizing these patients. A simplified approach, based not only on echocardiographic data but primarily on the symptoms and signs of HF, has been recently proposed [8]. Firstly, clinical features should be well investigated and distinguished from those conditions that may mimic this syndrome or contribute to its development, such as deconditioning, anemia, lung disease, and obesity. HFpEF is more likely to affect women and elderly people (aged > 60 years) with comorbidities, including arterial hypertension (AH), atrial fibrillation (AF) [13,14], diabetes mellitus (DM), and chronic kidney disease (CKD) [15,16]. Moreover, due to the wide variability in etiopathogenetic causes and presentation modalities, diagnosis and management are challenging, requiring a targeted and highly specific treatment [8].

Patients with HFpEF should be investigated in order for doctors to detect coronary artery disease (CAD) and arrhythmias, especially AF, HA, and valvular heart disease (VHD). Notably, an acute HF, dyspnea, caused by poor blood pressure control, volume overload, and/or tachyarrhythmias might represent the first clinical presentation [3]. Importantly, other conditions, despite being less common, should also be excluded in case of clinical suspicion of HFpEF, such as cardiomyopathies (CMP), infiltrative and storage disorders (sarcoidosis, hemochromatosis), endomyocardial, pericardial, metabolic and neuromuscular diseases’ toxin-induced CMP, post-myocarditis syndrome, and drug-induced HF. Particularly in the cardiac form of amyloidosis, prompt recognition of “red flags” may lead to early diagnosis and treatment [8,17].

Symptoms such as dyspnea, fatigue, ankle swelling, nocturnal cough, and wheezing and common signs such as peripheral edema, tachycardia, tachypnoea, hepatomegaly oliguria, and pleural effusion should be carefully detected in order to recognize and treat HFpEF early [8].

## 3. Diagnostic Tools

Echocardiography plays a pivotal role in diagnosing HFpEF and almost all aspects of its management [8]. Moreover, the majority of score-based algorithms to help clinicians in the diagnosis of HFpEF (such as H2FPEF and HFA-PEFF) [15,18] are based on echocardiographic parameters (left atrialLA size, mitral E velocity, septal e′ velocity, and E/e′ ratio) and can be obtained only by means of an accurate echocardiographic evaluation. Moreover, an echocardiographic exam is useful to monitor the status of LVEF in the case of a new onset of symptoms in subjects with already known HFpEF, although, in most cases, LVEF is likely to remain stable over the years. Undoubtedly, invasive cardiopulmonary exercise testing (iCPET) is the most accurate tool for diagnosing HFpEF. Pulmonary capillary wedge pressure (PCWP) cut-offs of ≥15 mmHg and ≥25 mmH at rest and during exercise, respectively, and an LV end-diastolic pressure (LVEDP) ≥16 mmHg at rest, measured through right-heart catheterization (RHC), have been considered to be diagnostic for HFpEF [3]. However, this kind of test is performed in a limited number of hospitals, so other tests are mainly used to make a diagnosis. Furthermore, cardiac magnetic resonance (CMR) is an emerging diagnostic tool for identifying patients with HFpEF. Indeed, due to its unique capability for myocardial tissue characterization and high-resolution imaging, it provides additional information and may be useful in phenotyping HFpEF and assessing subjects with HFpEF [19].

Notably, asignificant utility has been reported in distinguishing between acquired and inherited CMP that manifest as HFpEF, such as Fabry disease and amyloidosis. This aspect is crucial, given the existence of specific treatment options aimed at reversing or arresting these progressions [19].

The routine evaluation of B-type natriuretic peptide (BNP) and N-terminal fragments of proBNP (NT-proBNP) and natriuretic peptides (NPs), has been proposed for diagnosing and stratifying risk in HF [8].

Values of NT-proBNP, BNP, and mid-regional pro-atrial natriuretic peptide (MR-proANP) lower than125 pg/mL (or ng/L), 35 pg/mL, and <40 pmol/L, respectively, have been considered to be highly reliable in ruling out HF (negative predictive value of 95–99%) [8].

However, it has been shown that, in some conditions, such as increased androgenicity in women, insulin resistance, and in patients with particular genetic disorders or those treated with medications like spironolactone, NPs values can be normal or lower [20]. Moreover, NT-proBNP values have been reported to be significantly lower in obese patients with HFpEF than in those without obesity [21]. Indeed, NPs’ release is strictly linked to left ventricular (LV) wall stress. For this reason, NP dosage is more accurate in evaluating HFrEF than HFpEF [15].

Conversely, NPs levels may increase in several cardiac and non-cardiac conditions such as AF, CKD, and pulmonary disease and in advanced age [22].

Therefore, NPs are not ideal biomarkers in HFpEF assessment [6], especially in the chronic subset of patients, although they have an important prognostic significance [23]. Recently, new algorithms have been proposed to diagnose HFpEF [24], such as the H2FPEF score (without NPs) [18] or the HFA-PEFF score (including NPs and other criteria) [15].

Cardiac troponin (cTn) is another cardiac-specific biomarker that has been evaluated in several studies [25]; its prognostic significance in both acute and chronic HFpEF has been well established [25,26]. Particularly, higher cTn serum levels are associated with myocardial or microcirculatory dysfunction [25]. Furthermore, the heightened levels of hs-cTnT may be useful in distinguishing cardiac amyloidosis (CA) from HFpEF in light of the fact that, in CA patients, significantly elevated serum hs-cTnT levels have been reported compared to those with cardiac hypertrophy unrelated to CA. Indeed, it has been reported that CA accounts for 13% of HFpEF cases [27]

A recent study has shown that evaluating high-sensibilitycTn T, NT-proBNP, and eight other variables helps stratify morbidity and mortality [28].

Persistently elevated cTn levels in subjects with known risk factors may indicate the presence of HF stage B [29]. Furthermore, cTn assessment is recommended in acute HF to exclude acute myocardial infarction (AMI) [8]. Non-cardiac-specific biomarkers associated with inflammation and fibrosis (e.g., C-reactive protein, Interleukin-6, Interleukin-1β, soluble suppression of tumorigenesis 2, Galectin-3, matrix metalloproteases) have been recently proposed in clinical practice for the better management and treatment of acute and chronic HFpEF.

## 4. Comorbidities

### 4.1. Obesity

Obesity is one of the most frequent comorbidities among patients with HFpEF [30], increasing the risk of developing HFpEF over HfrEF. Indeed, obesity occurs in 80% of patients with HFpEF [31,32]. It has been shown to play an important role in the pathophysiologic mechanisms of HFpEF. Therefore, obesity-related HFpEF has been proposed as a peculiar phenotype with specific underlying mechanisms contributing to HF progression. These mechanisms include sodium retention, neurohormonal regulation disruptions, energy substrate metabolism changes, pulmonary hypertension, pericardial constraints, and systemic inflammation.

A body mass index (BMI) > 30 kg/m^2^ is usually used to define obesity [32]. However, the BMI has several drawbacks when assessing the association between obesity and HF. The BMI cannot evaluate body composition (e.g., proportion of muscle and fat mass). An intriguing phenomenon known as the “obesity paradox” has been observed among individuals with heart failure, wherein those who are mild-to-moderately obese tend to exhibit a more favorable prognosis compared to underweight patients [33]. This paradox can partly be elucidated by recognizing that a high BMI in certain individuals may correspond to a higher lean mass, a surrogate skeletal muscle mass indicator. This increase in lean mass can subsequently result in enhanced cardiorespiratory fitness and improved survival among patients with HFpEF [34]. Therefore, recent studies have focused on evaluating central obesity, measuring visceral adiposity with waist circumference, waist/hip ratio, or computed tomography (CT) [35]. Sarcopenic obesity, which is defined by an excess of adiposity, particularly central obesity, along with a decline in muscle mass, has been linked to increased hospitalizations, diminished QoL, and higher mortality rates in individuals with HFpEF. Conversely, obese patients who maintain physical fitness and retain their skeletal muscle mass tend to have a more favorable prognosis than those who are obese and sarcopenic [36]. For obese patients with HFpEF, it is advisable to consider caloric restriction and exercise training as part of the recommended management approach due to the fact that they have been shown to have positive and additive effects on exercise capacity and symptoms in a randomized clinical trial [37]. Bariatric surgery could be considered in selected patients [38].

Glucagon-like peptide 1 (GLP-1) receptor agonists are increasingly recognized for their potential in managing type 2 diabetes (T2DM) and obesity. These medications exhibit diverse effects, including stimulating insulin secretion based on glucose levels, delaying gastric emptying, and inhibiting postprandial glucagon secretion. Consequently, GLP-1 receptor agonists have demonstrated efficacy in controlling glycemia among T2DM patients and reducing serious CV events in patients with both T2DM and CV or renal disease. Moreover, these agonists have been shown to induce weight loss in obese subjects, irrespective of T2DM. Given these encouraging outcomes, GLP-1 agonists are under consideration for application in other obesity-related conditions.

In patients with HFpEF, those concurrently experiencing obesity appear to present more unfavorable clinical and hemodynamic features in comparison with their non-obese counterparts. Subjects with obesity-related HFpEF manifest higher degrees of volume overload, right-heart dysfunction and remodeling, and elevated systemic inflammation levels. Furthermore, it has been suggested that adipose tissue may contribute to the onset and progression of HFpEF. Consequently, weight loss achieved through lifestyle modifications and pharmacological interventions holds significant potential for improving outcomes in this patient population.

In the STEP-HFpEF trial [39], on 529HFpEF obese patients categorized according toLVEF, treatment with semaglutide significantly reduced symptoms and body weight compared to a placebo, regardless of LVEF. The encouraging findings support the use of GLP-1 agonists in treating obese HFpEF adults. Notably, the observed improvement in physical function indicates significant positive alterations in the QoL for individuals administered with semaglutide. Semaglutide, in light of these outcomes, warrants consideration as an adjunctive therapy for individuals with obesity-related HFpEF.

### 4.2. Diabetes Mellitus

Diabetes mellitus (DM) coexists in approximately 45% of patients with HFpEF [40], and its prevalence is higher in those with new-onset HFpEF [41].

Recently, patients with DM and HFpEF have been shown to have worse morbidity and mortality than those without DM [42,43,44,45,46].

**Figure 1:**
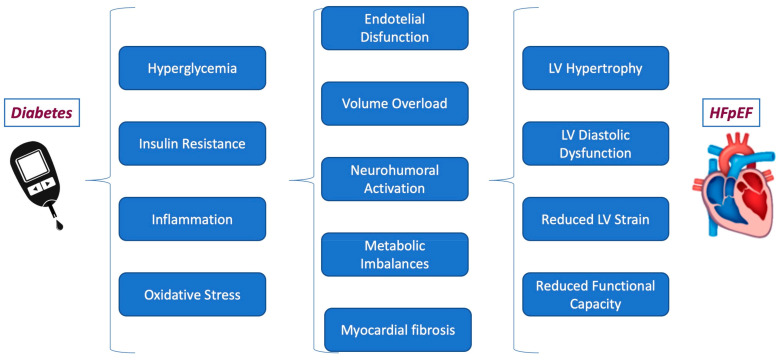
The pathophysiological mechanism linking diabetes to HFpEF.

Although still not fully understood, several pathophysiological mechanisms, such as volume overload due to impaired natriuresis, neurohumoral activation, and metabolic imbalances, have been hypothesized to contribute to the poorer prognosis of patients with DM and HFpEF [47] (Figure 1).

It has been well assessed that intensive blood glucose control fails to improve CV mortality or HHF in patients with DM but, instead, increases their predisposition to hypoglycemia [48]. In recent years, novel glucose-lowering treatments, especially SGLT-2i, have been demonstrated to reduce major adverse CV outcomes and HHF in patients with DM [49]. This has led to the introduction of these drugs in the management of DM patients at high CV risk, supported by recent guidelines [50]. Moreover, recent findings from the EMPEROR-PRESERVERD [51] and DELIVER [52] studies have highlighted that empagliflozin and dapagliflozin decreased the risk of CV death or HHF in patients with HFpEF, irrespective of whether they had DM or not [51].

Patients with HFpEF and diabetes have been shown to benefit from sacubitril/valsartan treatment in terms of a significant reduction in HbA1c compared to valsartan and a lower need for insulin [53].

Therefore, a team-based approach is essential in order to address the multiple comorbidities of patients with HFpEF and DM and, at the same time, decrease CV mortality and prevent hospital readmission.

### 4.3. Arterial Hypertension (AH)

AH, involved in the disease’s pathogenesis and prognosis, is the most commonly reported comorbidity in HFpEF [54].

**Figure 2:**
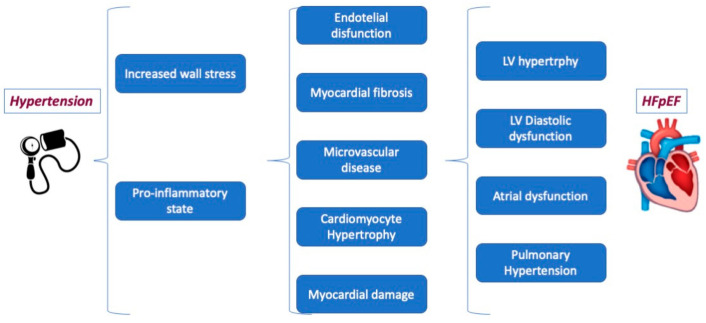
The pathophysiological mechanism linking arterial hypertension to HFpEF.

Furthermore, AH is considered to be the most significant modifiable risk factor for HFpEF through complex and multifactorial pathophysiological mechanisms, including left (not only)-ventricular (LV) hypertrophy and diastolic dysfunction (DD) but also coronary microvascular disease, endothelial dysfunction, myocardial damage, and fibrosis (Figure 2). Although no specific antihypertensive treatment has shown definitive mortality benefits, blood pressure (BP) control is commonly considered a cornerstone of HFpEF prevention and clinical care [55]. In patients with HFpEF and AH, specific treatment should be adopted [6,23]. Based on current evidence [8], the first treatment choices are angiotensin-converting enzyme inhibitors (ACE-i) or angiotensin II receptor blockers (ARBs), spironolactone, and diuretics. Non-pharmacological approaches, including lifestyle changes, sleep disorder treatment, and renal sympathetic denervation, may offer additional morbidity benefits in the HFpEF population [56].

Regarding pulmonary hypertension (PH), its prevalence in HF is widely variable, and, moreover, a complete understanding of its determining factors remains unclear [57].

The high left-sided filling pressure characterizing left-sided HF has been recognized to result in post-capillary PH or group 2 PH. When systolic function is preserved, the occurrence of PH is correlated with the severity of LV diastolic dysfunction [57]. This association has been demonstrated in individuals with aortic stenosis and a normal EF [57].

**Figure 3:**
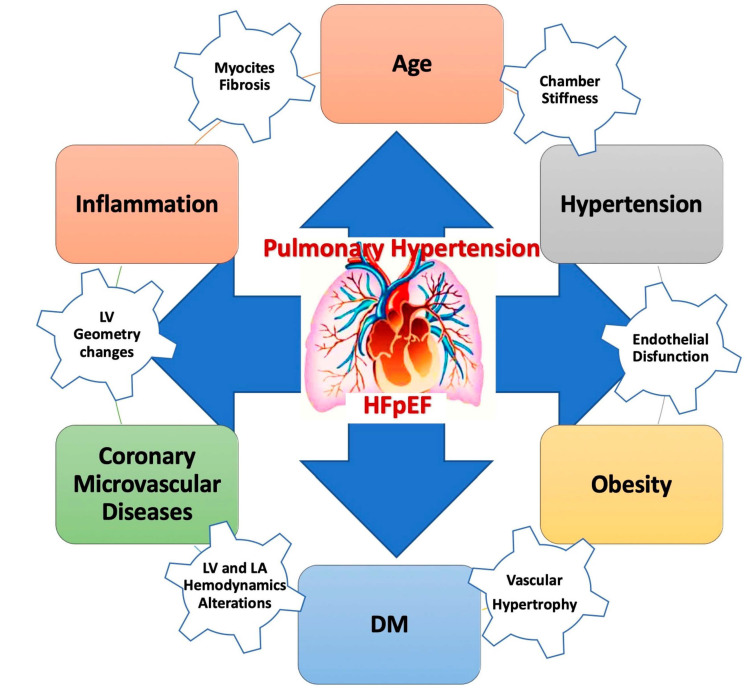
Pulmonary hypertension in HFpEF. DM: diabetes mellitus; HFpEF: heart failure with preserved ejection fraction.

In addition, reactive elevation in pulmonary arterial tone or intrinsic arterial remodeling leads to an additional pre-capillary PH. PH is known to be an important contributor to morbidity and mortality in patients with HFpEF [57] (Figure 3).

Right-heart catheterization (RHC) is considered the gold standard for investigating the hemodynamic response during exercise, distinguishing PH-HFpEF from pre-capillary forms of PH [58]. However, discriminating between these two forms is essential in order to diagnose and adequately manage these patients because the therapeutic strategies are completely different [59].

The presence of PH involves important clinical implications in HFpEF. Diagnosing and treating this condition is essential. The complex pathobiological mechanisms of HP in HFpEF are shown in the figure above.

### 4.4. Chronic Obstructive Pulmonary Disease (COPD)

Chronic obstructive pulmonary disease (COPD) is common among individuals with HFpEF, and it is linked to significantly more severe symptoms, reduced QoL, and lower long-term survival when compared to patients with HFpEF without COPD [60,61].

Patients with HFpEF and COPD exhibit an elevated presence of LV fibrosis and remodeling and present increased aortic stiffness and aortic pulsatile load compared to those with HFpEF alone [62].

**Figure 4:**
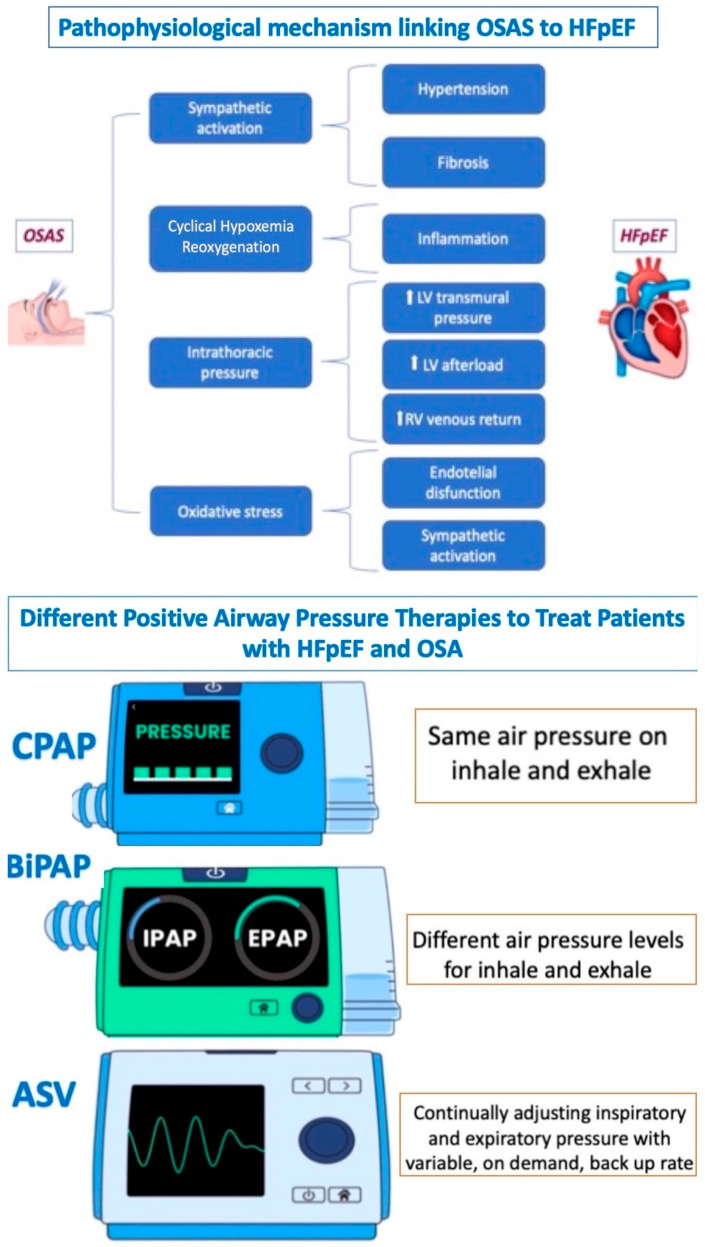
Relationship between OSAS and HFpEF. OSAS: obstructive sleep apnea syndrome; HFpEF: heart failure with preserved ejection fraction; PAP: continuous positive airway pressure; BPAP: bilevel positive airway pressure; and ASV: adaptive servo ventilation.

Symptoms and signs frequently overlap in these systemic conditions, making a differential diagnosis difficult. Echocardiography and pulmonary function tests should be performed on each patient, and the results should be carefully evaluated to avoid misdiagnosis and inappropriate treatment (Figure 4). Therefore, a great collaboration is needed between pulmonologists and cardiologists to identify and manage coexisting HFpEF and COPD appropriately [63].

HF and COPD guidelines recommend essential therapeutic interventions, including smoking cessation and influenza vaccinations [64,65].

Data from the DELIVER trial (which reported a decrease in the risk of worsening HF events or CV death in patients treated with dapagliflozin compared to the placebo group) reported that the coexistence of mild-to-moderate COPD was correlated with a more pronounced decline in health status and unfavorable clinical outcomes. Notably, dapagliflozin exhibited a consistent impact across patients with and without COPD, reducing the risk of deteriorating HF or CV death and maintaining safety, regardless of COPD status [66].

### 4.5. Sleep Apnea

Another frequently observed comorbidity in HFpEF is obstructive sleep apnea (OSA), which is found in nearly half of this population and is related to an increased arrhythmia burden and increased mortality [67,68,69]. For this reason, all patients with HFpEF should be screened for symptoms suggestive of OSA, such as excessive daytime sleepiness, episodes of stopped breathing during sleep, abrupt awakenings, morning headaches, difficulty concentrating during the day, etc. This assessment aids in determining whether further diagnostic assessments and/or therapeutic interventions are warranted.

OSA, as another cause of HFpEF, is associated with activating several pathophysiological pathways (inflammatory, metabolic, neural, and hemodynamic) that lead to the development of myocardial fibrosis and alterations in collagen, resulting in compromised ventricular function, particularly during physical exercise.

Recent studies support positive airway pressure (e.g., CPAP, bi-PAP, or ASV) as an effective method to treat patients with HFpEF and OSA, particularly in reducing symptoms and improving functional status [70,71,72] (Figure 4).

### 4.6. Chronic Kidney Disease

Individuals who have both HFpEF and chronic kidney disease (CKD) constitute a patient population with a less favorable prognosis. In this group, there is an increase in CV morbidity and mortality as renal function declines. Several inflammatory, neurohormonal, and hemodynamic pathological processes may contribute to developing both pathologies and represent common therapeutic targets [73].

CKD is characterized by a glomerular filtration rate (eGFR) < 60 mL/min/1.73 m^2^. Nevertheless, it is important to note that proteinuria or albuminuria can indicate impaired kidney function and may frequently manifest before a decline in eGFR occurs. Consequently, CKD is often underdiagnosed or underreported due to its reliance solely on eGFR measurements [74].

Acute kidney injury (AKI) is common among patients referred to the hospital with decompensated acute HFpEF. Currently, treatment options for HFpEF are limited, and they become even more constrained when CKD is present along with hyperkalemia, which is a significant concern in clinical practice [75].

Recently, the EMPEROR-preserved [51] and DELIVER [52] studies demonstrated the beneficial effect of SGLT2i in terms of reducing the combined risk of CV death or HHF in patients with HFpEF. Taking these results together with the demonstrated renal efficacy and safety of SGLT2i in terms of decreasing renal outcomes, these drugs seem to be promising for treating patients with HFpEF and CKD [52]. Moreover, two pre-specified analyses of the DELIVER [76] and EMPEROR PRESERVED [77] trials demonstrated that the treatment benefit of dapagliflozin and empagliflozin, respectively, in patients with HFpEF is not influenced by baseline kidney function. Furthermore, although treatment with SGLT2i in these trials did not significantly lower the frequency of the kidney composite outcome, it was significantly associated with a lower decline in eGFR compared to the placebo.

### 4.7. Sarcopenia

Sarcopenia is characterized by muscle wasting and compromised muscle mass, function, and strength [78]. The age-related decline in skeletal muscle mass and function constitutes a natural aspect of the aging process. However, chronic diseases like cancer and HF can pathologically accelerate this process. There are several methods to assess sarcopenia, but the most widely used are the Asian Working Group for Sarcopenia (AWGS) criteria [79]. Sarcopenia is frequently observed in HF, and its occurrence is notable in this patient group when compared to individuals of a similar age. This elevated prevalence is similarly consistent among patients with HFrEF and HFpEF [80,81]. It represents a major cause of exercise intolerance and ventilatory incompetence in patients with HF [82]. Muscle wasting facilitates the aggravation of other clinical conditions and harms QoL. Patients with sarcopenia have longer and more frequent hospitalizations and worse outcomes [83,84]. Sarcopenia can be modifiable; a multidisciplinary approach is warranted to manage it. Currently, the management of sarcopenia is based on two pillars: exercise training and dietary intervention. Physical activity improves functional status, peak VO_2_, and QoL among subjects with HFpEF [85]. As exercise training has shown limited effects on cardiac function among patients with HFpEF [86], it seems that the positive impact of training on exercise tolerance primarily stems from enhancements in peripheral skeletal muscle health and function.

Data based on patients with HFrEF and healthy elderly subjects showed several positive skeletal muscle adaptations after exercise training, including increased percent oxidative fibers, oxidative enzyme activity, and capillary density. Despite the lack of mechanistic studies among patients with HFpEF, similar effects could be hypothesized [87]. There is little evidence that exercise should be combined with a protein-rich diet (1–1.5 mg/kg/day) [88]. Despite small studies showing possible positive effects of drug therapies (e.g., testosterone, growth hormone), there are no sufficient data to recommend such treatments in this scenario [89].

### 4.8. Anemia and Iron Deficiency 

Anemia has been commonly described in the HFpEF population and has been related to poor outcomes [90]. The etiology of anemia in HF is multifactorial and may be due to hemorrhage (e.g., gastrointestinal bleeding), hemodilution, bone marrow deficiency, renal dysfunction, iron deficiency, inflammation, and nutritional and metabolic factors. Anemia may lead to decreased oxygen transport, thus reducing exercise tolerance and increasing sympathetic and renin–angiotensin–aldosterone systems’ activity. Hemoglobin values <12 g/dL and <13 g/dLin women and men, respectively, define the anemia condition. Iron deficiency (ID), which can be present independently of anemia, is [8] also frequent in patients with HFpEF and can be found in up to 60% of patients [91]. ID is associated with worse symptoms and impaired functional capacity in these patients, irrespective of anemia. ID in HF is identified by ferritin values < 100 μg/L (absolute ID) or ferritin 100–300 μg/L combined with transferrin saturation (TSAT) < 20%) [8]. Regular screenings for anemia and ID in all patients with HF have been recommended [8]. This screening should involve a comprehensive evaluation, including a complete blood count, serum ferritin concentration measurement, and TSAT determination [8]. When anemia or ID is documented, further investigation should be conducted to define the cause. Erythropoiesis-stimulating agents (e.g., darbepoetin-alpha) to correct anemia in HF have not been shown to improve mortality and have instead raised safety concerns by increasing the risk of thromboembolism. Therefore, these agents are not recommended in HF [8,92]. Several studies have documented that ID in patients with HF can be corrected with intravenous iron (ferric carboxymaltose) to improve symptoms, exercise capacity, QoL, and outcomes [93]. However, intravenous iron replacement therapy has largely been studied in the HFrEF population; no data are available for HFpEF. The AFFIRM-HF trial [94] showed a reduction in hospitalizations due to iron replacement therapy in patients stabilized after an acute HF event. One-third of the patients enrolled had an LVEF between 40 and 50%. Therefore, it is plausible that ID may play a role also in the pathogenesis and prognosis of HFpEF and that treatment with intravenous iron therapy may be effective in patients with HFpEF. Two ongoing randomized clinical trials are evaluating the effect of intravenous iron replacement therapy in patients with HFpEF: the FAIR-HFpEF (NCT03074591) and the PREFER-HF (NCT03833336) trials. Oral iron therapy was found to be ineffective in replenishing iron levels. Furthermore, it did not improve exercise capacity among patients with HFrEF who also had ID, probably due to the reduced absorption of oral iron in these patients and increased hepcidin levels. Therefore, oral iron therapy is not recommended for treating ID in patients with HF [95].

### 4.9. Depression

Depression has a prevalence of 20–30% among patients with HF and is notably correlated with a diminished QoL and adverse clinical outcomes [96]. Patients with HF should be screened for depressive symptoms when there is clinical suspicion, using a validated questionnaire [8]. The Beck Depression Inventory and Cardiac Depression Scale are tools formally validated for assessing depressive symptoms in patients with HF. Patients with severe depressive symptoms in the questionnaires should be referred for further evaluation.

The presence of depressive symptoms does not necessarily correspond to the diagnosis of depression, which should be made after a clinical interview by a specialist. The best management of depression among patients with HF is still debated. Exercise therapy may positively affect symptoms and outcomes in both HF and depression. Cognitive behavioral therapy has been found to improve depressive symptoms but has little effect on HF symptoms [97]. Tricyclic antidepressants should not be chosen for treating depression in HF, considering the fact that there is a higher risk of worsening HF and arrhythmias. Selective serotonin reuptake inhibitors (SSRI) have been shown to have conflicting results in improving depressive symptoms and neutral effects on outcomes [96]. Most data about depression in HF are focused on patients with HFrEF, while only a few data are available for patients with HFpEF. Higher baseline depressive symptoms and worsening depressive symptoms have been related to all-cause mortality in patients with HFpEF [98]. Exercise training in HFpEF showed neutral results on depressive symptoms [99], and no data about antidepressant drugs in HFpEF are available. Interestingly, spironolactone has been associated with a modest reduction in depressive symptoms in the TOPCAT trial [98]. Further studies are needed to evaluate the mechanisms underlying potential treatments of depression in the context of HFpEF.

## 5. Evidence-Based Medicine: Appropriateness of Treatment and Patients Taking Charge

### 5.1. Angiotensin-Converting Enzyme Inhibitors (ACE-I)

As previously reported, a maladaptive activation of the renin–angiotensin–aldosterone system (RAAS)is involved in the continuum of disease development from HA to HF. Since this treatment has been demonstrated to improve prognosis in patients with both AH and HFrEF, it has been hypothesized that ACEi and other drugs counteracting the RAAS system might also positively impact HFpEF. However, data from RCTs did not confirm this assumption.

The Perindopril for Elderly People with Chronic Heart Failure trial (PEP-CHF) randomized 850 patients to placebo or perindopril over a median follow-up of 2.1 years [100]. The enrolled patients had a median LVEF of 64% and a high prevalence of AH (79%) and diabetes mellitus (21%). This study showed no difference in the primary composite outcome of death and HHF between perindopril and the placebo.

However, the patients randomized to perindopril had improvement in symptoms and exercise capacity.

For these reasons, ACE-I is not suggested as a disease-modifying therapy in the latest ESC guidelines. However, this therapy is suggested as a treatment in hypertensive patients to prevent or delay the onset of HF [8].

### 5.2. Angiotensin Receptor Blockers (ARBs)

ARBs counteract the effect of angiotensin II, mimicking the same benefit related to ACE-I treatment. Also, this drug class has been thought to be effective in patients with HFpEF.

In the CHARM-Preserved trial, carried out on more than 3000 HF adults with LVEF > 40% and a high prevalence of AH (64%) and DM (28%) [101], CV death and HHF were not reached in the candesartan group compared tothe placebo. However, there were fewer HHF in the candesartan group, with a significant difference from the placebo [101].

In the I-Preserve [102] trial on 4128 adults with LVEF > 45% randomized to a placebo or irbesartan, there was no difference between the two treatments for the primary outcome (death for all causes or CV hospitalization for CV causes). Moreover, other pre-specified outcomes, such as variations in NPs or the QoL scores, did not change at six months.

As well as ACE-I treatment, ARB therapy is also suggested in the latest ESC guideline to treat hypertension, with the aim of preventing the development of HF or HF-related symptoms [8].

### 5.3. Beta-Blockers

The mechanism related to the potential benefit of beta-blockers in patients with HFpEF could be related to counteracting hypertrophy and DD [103]. In this regard, some observational studies confirmed these data, although this benefit was not translated into reduced clinical outcomes in RCTs.

Two RCTs assessed the role of nebivolol [104] and carvedilol [105] in patients with chronic HFpEF. In the SENIOR trial on 752 patients with LVEF > 35% treated with nebivolol vs. placebo, the all-mortality or CV hospitalization rates did not decrease [104]. Similarly, in the J-DHF trial, including 245 patients with LVEF > 40% randomized to a placebo or carvedilol, no difference in CV mortality or HHF was reported [105].

Recently, a meta-analysis of RCTs, which investigated the role of beta-blockers across all spectrums of HF phenotypes, showed that this treatment was ineffective in reducing the all causes of death or CV mortality rates in patients with LVEF > 50% [106] (Supplementary Materials Table S1). The lack of effectiveness was consistent in patients with sinus rhythm and AF.

### 5.4. Sacubitril/Valsartan 

In the context of HFpEF, sacubitril/valsartan has been tested in numerous studies. The PARAMOUNT trial [107] was a phase-2 RCT with 36 weeks of follow-up comparing sacubitril/valsartan with a placebo in 200 subjects. A significant decrease in pro-BNP was found, with a good overall safety profile.

Subsequently, 4796 patients were randomized in the phase-3 PARAGON-HF RCT [2]. The primary composite endpoint of CV death and total HHF was tested in a 35-month follow-up. Although a benefit in reducing the primary endpoint was observed (relative risk 0.87), this was not statistically significant. Only the secondary renal outcome (reduction in GFR > 50%, development of end-stage renal disease (ESRD), or death attributable to renal causes) showed a significant benefit in favor of sacubitril/valsartan [108].

Similarly to PARADIGM-HF (the trial on HFrEF), the sacubitril/valsartan-treated patients experienced more frequent hypotension (15.8 vs. 10.8%, *p* < 0.001) and angioedema, with less hyperkalemia occurrence and discontinuation of study medication due to adverse events when compared with the valsartan-treated patients.

Important data come from pre-specified subgroups and post hoc analyses. The benefit of primary composite outcome reduction became significant in the lower range of EF (45–57%) compared to those with higher values [108], and women had a better outcome than men [109]. Similar results were also confirmed when data from PARADIGM-HF and PARAGON-HF populations were pooled together [110]. However, there were no differences in the treatment effect regarding symptoms and QoL according to sex. Thus, the authors did not explain the observed better prognosis in women.

An important post hoc analysis of the above-mentioned trial was related to a benefit assessment, dividing patients in terms of time from a previous hospitalization [111]. Indeed, the absolute risk reductions linked to sacubitril/valsartan were more conspicuous among patients who were enrolled in the trial shortly after hospitalization, with reductions of 6.4% (within 30 days), 4.6% (between 31 and 90 days), and 3.4% (within 91 to 180 days). Conversely, no discernible risk reduction was observed in patients randomized after more than 180 days or never hospitalized. Since 52% of the enrolled patients have never been hospitalized, this probably drives the absence of a significant benefit in the whole-group analysis.

How important the duration of HF is has been evaluated in a recently published post hoc analysis of PARAGON-HF RCT [112], which reported that the longer the HF duration was, the higher the risk of HFH in the absence of significant variations in the effects of sacubitril/valsartan. Moreover, the highest benefit was reported in those subjects with long-standing HF.

In the PARAGON-HF trial, on 4796 patients with HFpEF randomly assigned to receive either sacubitril/valsartan or valsartan, it was revealed that higher baseline levels of high-sensitivity troponin-T (hs-TnT) were linked to an increased risk of CV death and total HHF. Conversely, a reduction in hs-TnT levels at week 16was associated with a subsequent decrease in the risk of CVD/HHF compared to individuals who maintained consistently elevated hs-TnT values. Notably, the use of sacubitril/valsartan treatment resulted in a substantial reduction in hs-TnT levels compared to the use of valsartan alone. These findings suggested that measuring hs-TnT could be a valuable tool in identifying those patients with HFpEF who are more likely to benefit from sacubitril/valsartan therapy [113].

The latest study with sacubitril/valsartan in HFpEF was the PARALLAX-HF trial [7], a 24-week-long study with pro-BNP levels, distance walked at the 6 min walking test(6MWT), NYHA class, and KCCQ score as the endpoints. Despite an improvement in natriuretic peptides, no benefit was shown in the other endpoints. Since the mean EF was 56% (57% in PARAGON-HF), with only 19% of the subjects having values lower than 50%, only 35% of the subjects had a prior hospitalization, and there were fewer subjects (2566) than in PARAGON-HF, the results could not be substantially different from the latter trial.

The PARAGLIDE-HF trial [114] was recently published with the enrollment of 466 patients with HFpEF and a recent (<30 days) HHF or ambulatory intravenous diuretic use. Despite a significant improvement in natriuretic peptides, no significant improvement in cardiovascular death or HFH was observed in the eight months of follow-up, despite a significant trend in favor of sacubitril-valsartan. The limited patient population and the brief duration of the follow-up are likely to have influenced the outcomes of this clinical trial. Indeed, when the PARAGLIDE patients were analyzed together with those with similar characteristics (<30-day HFH or diuretic use) from the PARAGON HF trial [115] (participant-level pooled analysis), a significant benefit on all-cause death and HFH was observed, particularly in those with an EF lower than 60% (RR 0.78; 95% CI 0.66–0.91) [116].

The results of the four main trials on sacubitril/valsartan and HFpEF were recently meta-analyzed [117], with further confirmation of the overall positive effects of this treatment on the composite outcome of all-cause CV death and HHF.

Two imaging studies were published in 2023. A post hoc analysis of the PARAMOUNT trial [118] was recently published, showing a benefit on the global circumferential strain determined by sacubitril/valsartan treatment, without significant changes in the global longitudinal strain. These results are probably determined by the fact that circumferential strain was more impaired in the study compared to the longitudinal one, as found in other HFpEF echocardiographic studies [119]. Another study using cardiac magnetic resonance (PARABLE) [120] demonstrated a significantly higher increase in left atrial volume with sacubitril/valsartan, probably reflecting an increase in vascular compliance.

All these results led the FDA to approve sacubitril/valsartan also for HFmrEF and HFpEF (label extension obtained in February 2021). Despite this, the prescription of sacubitril/valsartan in this setting is still under-represented [121]. No other national pharmacological regulatory agency worldwide has extended the prescription to HFpEF. Supplementary Materials Table S2 summarizes the main significant study results with sacubitril/valsartan in HFpEF.

In summary, although the main HFpEF study with sacubitril/valsartan [53] (PARAGON-HF) did not show significant results regarding the primary composite endpoint, some subgroups, such as HFmrEF patients (LVEF 45–57%), those with a recent HHF hospitalization, and women, have been shown to be more likely to benefit from this treatment.

### 5.5. Mineralocorticoid Receptor Antagonists (MRAs) and Diuretics

Mineralocorticoid receptors are expressed in a wide variety of cell types, including renal tubular epithelial cells, endothelial cells, cardiomyocytes, cardiac fibroblasts, vascular smooth muscle cells, adipocytes, and immune cells.

Uncontrolled mineralocorticoid receptor activation may contribute to detrimental mechanisms such as inflammation, oxidative stress, interstitial and perivascular fibrosis, and insulin resistance, leading to CV and renal damage [122,123,124].

The use of steroidal mineralocorticoid receptor antagonists (MRAs), spironolactone, and eplerenone has been considered an effective therapeutic approach for patients with HfrEF, enhancing their outcome.

On the contrary, MRAs treatment in HFpEF is not clearly suggested in the latest guidelines [29].

The aldo-DHF trial enrolled 422 ambulatory patients affected by HFpEF (NYHA class II or III), showing that 25 mg of spironolactone once daily versus a placebo improved LV diastolic function, evaluated using the E/e’ ratio, without influencing functional status [125].

In the TOPCAT randomized trial [85], which involved 3445 symptomatic patients with LVEF ≥ 45%, the primary composite outcomes (CV death, aborted cardiac arrest, or HHF) did not significantly differ between spironolactone and the placebo.

However, it is worth noting that, among the primary outcome components, only the incidence of hospitalization for heart failure showed a notable decrease in the spironolactone group compared to the placebo group (206 patients (12.0%) versus 245 patients (14.2%); hazard ratio, 0.83; 95% confidence interval, 0.69 to 0.99, *p* = 0.04). Spironolactone did not lead to a significant reduction in either total deaths or hospitalizations for any reason.

Nevertheless, it is important to mention that the treatment group experienced higher rates of hyperkalemia and elevated creatinine levels as the observed adverse effects [126].

The administration of MRAs may also carry the risk of adverse effects. Hyperkalemia is a common concern, particularly when steroidal MRAs are employed concurrently with another agent which inhibits the RAAS. Moreover, progestogenic and antiandrogenic adverse effects have been described, including gynecomastia, impotence, and menstrual irregularities [124,127]. These concerns about potential adverse events contribute significantly to the underutilization of MRAs in treating HF [128].

Nevertheless, the beneficial effects of MRAs in HFmrEF and HFpEF remain debated, requiring further investigation. The SPIRIT-HF (NCT04727073) and SPIRRIT-HFpEF (NCT02901184) clinical trials should confirm the potential use of spironolactone within these patient subsets [128].

In this field, a new class of non-steroidal MRAs including apararenone, balcinrenone, esaxerenone, finerenone, and KBP-5074 has recently undergone extensive preclinical investigation [128].

Finerenone is a highly selective non-steroidal MRA that acts as a classical MRA, like spironolactone, but without steroid-induced side effects such as gynecomastia. A protective effect of finerenone has been demonstrated in several preclinical animal models of different CV conditions (cardiac hypertrophy, atrial fibrosis, blood pressure, and arteriosclerosis) [129].

Finerenone has been shown to reduce new-onset HF and improve other HF outcomes, including the risk of first HHF and the rate of total HHF, in patients with CKD and type 2 DM, irrespective of a history of HF [130]

FINEARTS-HF is an ongoing randomized phase-III trial aimed at assessing the superiority of finerenone over a placebo in reducing mortality and total HF events. Its results are expected for 2024 (NCT04435626).

Additionally, the ongoing phase-II clinical trial MIRACLE (NCT04595370)NCT04595370aims to investigate the effectiveness and safety of another MRA, balcinrenone AZD-9977, comparing its administration alone to its use coupled with dapagliflozin in patients with HFpEF with an LVEF < 60% and concomitant CKD (Table 1).

Diuretics in HFpEF aim to attenuate symptoms and signs of congestion and maintain an appropriate fluid status [8]. Dosage should be adjusted over time according to volume changes in order to reduce potential adverse effects.

Loop diuretics (LD) should be preferred, although, in the presence of AH, a condition which commonly occurs in these patients, thiazide diuretics (TD) may be chosen.

Although this class of drugs is largely used in HFpEF, their effects on long-term prognosis remain unclear.

Although a lower risk of hospitalization by 30 days has been reported in patients treated with LD in a retrospective single-center study, these results were confirmed by an analysis of the TOPCAT trial, which otherwise revealed a non-significant reduction in all-cause hospitalizations related to LD use. Moreover, an interrupted LD regimen compared to a continuative regimen has shown no differences in terms of congestion makers [131].

Nevertheless, ventricular diastolic filling, orthostatic tolerance, and estimated GFR improved only in the withdrawal group of the above-mentioned study [132].

### 5.6. Sodium-Glucose Cotransporter-2 Inhibitors (SGLT2i)

In patients with HFpEF, most pharmacological treatments have limited or no impact on key outcomes, including QoL, functional status, hospitalization, and mortality. However, recent randomized clinical trials that tested SGLT2i in patients with HFpEF have shown that these drugs effectively impact clinical outcomes even in HFpEF (Table 2).

In the PRESERVED-HF trial, dapagliflozin significantly improved symptoms and self-reported and objectively measured physical limitations in patients with HFpEF with and without DM. These clinical benefits were observed after 12 weeks of treatment [133]. Following these encouraging data on the symptomatic relief provided by the use ofSGLT2 inhibitors in patients with HFpEF, more robust evidence has been obtained with two clinical trials that assessed the effect on harder endpoints, including hospitalization and cardiovascular mortality.

The EMPEROR-Preserved [51] and the DELIVER [52] trials have shown that both empagliflozin and dapagliflozin significantly reduce the combined endpoint of hospitalization or urgent visits due to HF and CV death (−21% and −18%, respectively). In both trials, the clinical benefit was mainly due to a lower HF hospitalization risk.

Of note, in the SOLOIST-WHF trial, even sotagliflozin (an inhibitor of both SGLT2 and gastrointestinal SGLT1), tested in patients with DM and a recent hospitalization for decompensated HF, has been found to improve outcomes (−23% hospitalization and CV rate) both in patients with HFrEF and in patients with HFpEF [134].

Ina pre-specified analysis including 11,007 subjects with HF with different ranges of LVEF from two trials [52,135], dapagliflozin has been demonstrated to reduce the risk of CV significantly and the total HHF irrespective of LVEF [136].

These results pave the way for a wider clinical use of SGLT2i, even in patients with HFpEF. The 2022 American guidelines state that SGLT2i can effectively reduce HHF and CV mortality (class II B) [29]. Moreover, in light of two trials with empagliflozin and dapagliflozin [51,52], the latest European recommendations for patients with HFpEF were updated in 2023, indicating a class-I level of evidence A for the use of an SGLT2 inhibitor (dapagliflozin or empagliflozin) in patients with HFpEF to lower HHF or CV mortality risk [2].

**Table 2:** RCTs on SGLT2i use in HFpEF.

	N° pt	DM	Follow-Up (Median)	Age	Sex (% Female)	LVEF	Treatment	Primary Outcome	HR; (95% CI)
**EMPEROR-PRESERVED [51]** **NCT03057951**	5988	with or without DM	26.2 months	71.8	44.6%	54%	Empagliflozin	CV deaths HHF	0.79; (0.69–0.90I) *p* <0.001
**PRESERVED-HF [133]** **NCT03030235**	324	with or without DM	3.0 years	70.0	57%	60%	Dapagliflozin	KCCQ-CS at 12 weeks after treatment initiation	68.6; (66.2, 71.0) *p* < 0.001
**DELIVER [52]** **NCT03619213.**	6263		2.3 year	71.8	43.6%	54%	Dapagliflozin	Composite of worsening HF or CV death	0.82; (0.73–0.92) *p* < 0.001
**SOLOIST-WHF [134]** **NCT03521934.**	1222	with DM and recent HF worsening	9.0 months	70	32.6%	20% with LVEF > 50%	Sotagliflozin	Total number of CV deaths HHF HF urgent visits	0.67 (0.52–0.85); *p* < 0.001
**EMPERIAL-Preserved NCT03448406** **[137]**	315	with or without DM	12 weeks	73.5	43.2	53.1	Empagliflozin	-6MWTD change in week 12	*p* = 0.37
**CHIEF-HF** **NCT04252287 [138]**	476	with or without DM	2 weeks	63.4 ± 13.3	45%	50% (60%)	Canagliflozin (100 mg)	Change in KCCQ TSS at 12 weeks	100 mg of canagliflozin or placebo
**VERTIS CV** **NCT01986881 [139]**	8246	with DM	3.5 years	64.4	30%	1007 patients with LVEF > 45%	Ertugliflozin	CV death, nonfatal MI, nonfatal stroke	0.97 (0.85–1.11) *p* < 0.001
**SCORED [134].**	10,584	with DM, CKD	16 months	72	40%	>45%	Sotagliflozin	risk of the composite of CV deaths, HHF	0.95 (0.86–1.05)
**DECLARED-TIMI-58 [140]**	17,160	with DM and CVD or multiple CVD risk factors	4.2 years	72	37%	1316 patients with 55%	dapagliflozin	Primary safety outcome ◦ Non-inferiority to placebo with respect to MACE Primary efficacy outcomes: ◦ MACE: CV death, MI, or ischemic stroke ◦ CV death or HHF	Primary safety outcome: *p* < 0.001 for noninferiority Primary efficacy outcomes: **MACE** 0.93; 0.84–1.03; *p* = 0.17 **CV death or HHF** 0.83; 0.73–0.95; *p* = 0.005)
**MUSCAT-HF** **NCT03315143 [141]**	160 Luseoglifloz(82) Voglibose (83)	DM and HF	12 weeks			>45%	Luseogliflozin or voglibose	Difference in change in BNP from baseline to 12 weeks between the patients receiving luseogliflozin and those receivingvoglibose	0.93; 0.78–1.10; *p* = 0.26
**CANDLE** **NCT03315143 [142]**	253	DM and stable HF	24 weeks	68	25%	≥50% (71%)	canagliflozin 100 mg or glimepiride	Percentage change (post/pre −1) from baseline in NT-proBNP at week 24.	0.48; −0.13–1.59, *p* = 0.226

LVEF: left ventricle ejection fraction; DM: diabetes mellitus; CKD: chronic kidney disease; HHF heart failure hospitalizations; MACE: major adverse cardiovascular events; BNP: brain natriuretic peptide; and KCCQ TSS: Kansas City Cardiomyopathy Questionnaire Total Symptom Score.

### 5.7. Education, Awareness, and Patient Self-Care

**Figure 5:**
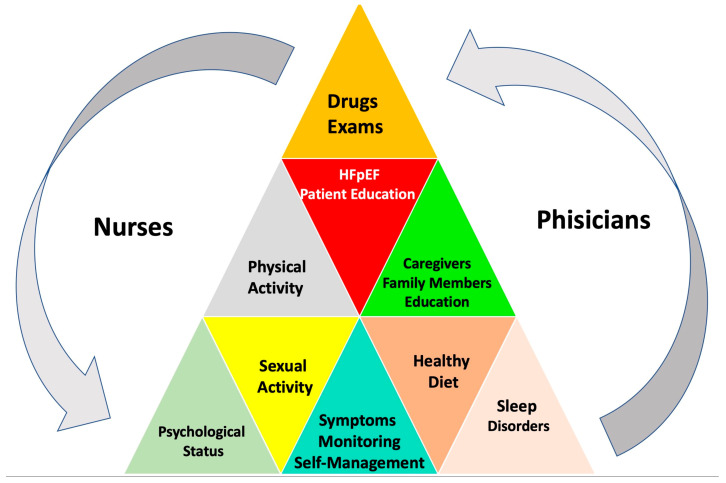
Patient education and self-support.

Providing adequate and up-to-date education regarding lifestyle based on scientific evidence, when available, or expert opinions is inextricably linked to an individual approach to chronic diseases [143]. Patient education is a key component of the multidisciplinary approach, which should be performed at the time of HF diagnosis, at discharge, and ideally before it [8]. Indeed, an appropriate counseling program allows patients to understand the management plans’ advantages and agree to self-monitoring [144]. As shown in Figure 5, education should consider comorbidities or communication barriers, including social failure and cognitive impairment.

The contents of health education include a basic HF overview, signs and symptoms along with their causes and consequences, lifestyle modification, risk factor control, adequate exercise, limiting diet and liquids, psychosocial aspects, implanted devices, medications (and their side effects), and the importance of adherence, regular checks, triggers for contacting a provider, where to obtain assistance and what caregivers need to know for the self-management of patients with HF [8,143,145,146,147,148].

HF educational interventions are not standardized and can vary in intensity, methodology, or strategy. The best form of education is unclear.

General educational strategies encompass the following components [8]:

1. Disseminating information using diverse formats considering the audience’s educational level and health literacy.
2. Employing group or face-to-face methods across one or more sessions, involving the active participation of both patients and caregivers. Several emotional techniques can be beneficial in this context.
3. Consistently reinforcing key messages at scheduled intervals, including the possibility of follow-up via telephone communication.

Motivational interviewing focuses on building a trusting relationship with the patient and learning about the problems by inquiring about the patient’s main symptoms, subjective feelings, lifestyle, disease control experience, psychology, and difficulties throughout the course of the illness, seeking out solutions for self-care problems, encouraging patients to talk about their difficulties when changing behaviors, and guiding them to think about ways of solving these problems. Chen et al. demonstrated that the self-care behavior of HF subjects could be improved effectively through motivational interviewing [149,150]. Developing awareness of HFpEF consists in acquiring the ability to recognize functional impairment related to the disease. This process involves the patient’s recognition of their condition and the associated symptoms, abilities, behavior, functional deficits, cognitive impairment, and changes in relationships. An essential part of a patient’s awareness entails identifying changes and how to deal with their condition, maintaining a sense of identity, and managing the positive and emotional aspects of the disease. Patient self-care has been shown to be crucial in developing self-care skills [146]. The ultimate goal of patients with HF is to acquire skills necessary to perform daily activities; in other words, to minimize repeated symptom exacerbations and prevent the deterioration of the QoL of patients, it is necessary to improve their ability to recognize symptoms and manage HF [145,146,151].

The figure above underlines the importance of checking blood pressure, urine volume, blood glucose, body weight control, physical activity, sleep, psychological conditions (prompt detection of depressive symptoms or anxiety), and medications and observing changes in signs and symptoms, caregiver support, and patient and family education.

### 5.8. Adherence to Drugs and Lifestyle Advice

Treatment adherence has been defined as “the extent to which individual behaviors (such as medication, diet, lifestyle changes) are following recommendations accepted by a health care provider”. Adherence to a treatment regimen refers to the activities that individuals perform in order to maintain life, healthy functioning, personal growth, and well-being. In particular, adherence to pharmacotherapy is a key element of achieving adequate outcomes.

The complexity of strengthening medication adherence has been recognized over the last few decades [1]. A shift from a paternalistic physician–patient relationship has occurred in practice toward shared decision-making concepts. Patient and physician establish a partnership and a level of confidence and stable communication to select the right treatment [2]. Although medication and lifestyle adherence as a critical self-care behavior is necessary for maintaining physiological stability, reducing cardiocirculatory burden, reducing symptom burden, and increasing survival [3], it is not observed in 50% to 62% of patients with HF [4]. For instance, assessments of compliance with RAAS inhibitors and β-blockers among patients with heart failure exhibit significant fluctuations, from 40% to over 90%. These fluctuations are contingent upon the approach employed for measuring adherence, the monitoring period’s duration, and the patient group’s specific attributes [5]. In addition, HFpEF often coexists with cardiac or non-cardiac comorbidities [6]. Comorbidity is a significant issue that complicates HFpEF management, including symptom management and medication adherence [7]. Increased complexity in the treatment regimen leads to poor adherence among people with HFpEF. Hence, treatment regimen adherence is raised as a complex and challenging phenomenon for the individual and treatment team in managing HFpEF.

Unfortunately, poor adherence to evidence-based medications is associated with a fourfold increase in HHF, increased healthcare costs, morbidity, and mortality [8], and exacerbation of or more significant HF symptom burden [9]. In addition to the adherence problem, medication persistence is defined as “the duration from initiation to discontinuation of therapy”, and how long a patient stays on medication without a specified length of permissible treatment gap should be determined [10]. This distinction allows for the differentiation of “how well” patients take their medication from “how long” they take it for. In several studies, poor drug adherence or non-persistence in patients with HF, including HFpEF, have demonstrated increased morbidity, mortality, and healthcare costs [11].

Supportive family, positive personal characteristics, and health literacy were identified as subthemes of the driving forces behind treatment adherence in people with HF. On the contrary, factors such as negligence, psychological problems, cultural, social, and economic problems, physical limitations, and lack of self-care strategies were identified as subthemes of the deterrent forces behind treatment adherence in people with HF. Paying attention to the psychological problems of people with HF, using the family potential in treatment adherence, and increasing awareness about medications and dietary restrictions can improve treatment adherence and clinical outcomes in people with HFpEF [12].

Thus, due to the special importance of HF management, healthcare providers should pay special attention to the issue of treatment adherence. Urgent efforts are needed to improve the implementation of HF drug treatment with decision support for clinicians and patients and ensure broader access to structured multidisciplinary care, particularly addressing adherence to multiple drug therapies and lifestyle advice.

### 5.9. Educational Tools

It has been shown that the use of educational tools may be very useful in the management of patients with HFpEF. Diaries for recording daily weight and symptoms, informative booklets, brochures, and audiotapes can improve patient self-management.

Moreover, novel educational tools, including tablets, mobile phone applications, and interactive audio-visual programs, may provide individualized education [143,147,150,152,153,154,155,156].

In I-CARE, the role of nurses, cardiologists, and physiotherapists in managing patients with HFpEF has been highlighted.

However, it has been shown that multidisciplinary teams, including geriatricians, diabetologists, dieticians, psychologists, nurses, electrophysiologists, interventional cardiologists, cardiac surgeons, endocrinologists, nephrologists, pneumologists, and physiotherapists, incorporating lifestyle-directed interventions, patient education, and self-care support, may optimize therapy, improve outcomes, and reduce HHF readmission.

It is essential to correctly diagnose HFpEF by recognizing symptoms such as dyspnea and exertional intolerance, excluding alternative diagnoses [143,155].

Family members and caregivers play a crucial role in the management of patients with HF, affecting patients’ self-care. Consequently, caregivers’ knowledge about the disease and self-care activities should constantly improve.

It has been shown that those patients with HF who are involved in effective self-care tend to experience a higher QoL and lower readmission and mortality rates [8].

Formal education and support interventions have been associated with a 39% decrease in readmissions and hospital readmission costs [148,157] and a lower hospitalization time [155].

In the I-CARE program, a greater reduction in mortality has been reported in educated patients compared to non-educated ones. However, the non-educated patients were older, more often female, and with a more severe disease [158].

The effects of education cannot be evident in the short term. In a study by Majd et al., educational intervention resulted in a significant reduction in death among patients with HF in a long-term follow-up [150].

Furthermore, Hwang et al. observed no intervention effects in patients with depressive symptoms [159].

A lower mortality in patients involved in an education program has been reported in the ODIN cohort [147]. Moreover, other goals, including MBI, 6MWT, cholesterol values [160], anxiety, and QoL, have significantly improved in patients provided with educational interventions [157]. The MIGHTy-Heart trial confirmed the effectiveness of telehealth tools in the management of patients with HF [153].

## 6. Conclusions

HFpEF is a complex syndrome with an incidence consistently rising due to advanced age and a higher prevalence of the associated burdens of obesity, sedentary lifestyles, and cardiometabolic disorders. Recently, various definitions have been proposed, varying in their diagnostic approach, sensitivity, and specificity. In the latest European guidelines, it is claimed that HFpEF is characterized by HF symptoms and signs in the presence of cardiac structural and/or functional abnormalities (LV diastolic dysfunction/increase in LV filling pressures, high natriuretic peptides levels in patients with LVEF ≥ 50%, HF symptoms and signs) [2]. According to the 2022 AHA/ACC/HFSA guidelines [29], the threshold for HFpEF has been defined as an LVEF ≥ 50%. Similarly, in the 2023 ACC Expert Consensus, HFpEF is defined as signs and symptoms of HF with left ventricular EF (LVEF) ≥ 50% [4]. Nevertheless, the need for a unitary definition has become mandatory, considering that HFpEF is a chameleonic syndrome due to its wide range of clinical manifestations and the importance of excluding differential diagnoses.

Despite the recent advancements in comprehending its pathophysiological mechanisms as well as the recent introduction of novel pharmacologic and lifestyle-based approaches capable of enhancing the clinical status of patients and reducing morbidity and mortality, HFpEF remains insufficiently acknowledged in routine clinical practice. Diagnosing HFpEF is a considerable challenge, particularly in a chronic setting.

Recent studies highlight the crucial role of an individualized approach in enhancing the phenotypic characterization of this disease and customizing treatment more effectively.

Additionally, it is recommended that all patients consider the implementation of exercise and lifestyle adjustments, particularly to facilitate weight reduction. Adopting team-based management involving a pool of healthcare professionals to optimize patient care, especially for those with multiple comorbidities, is an emerging need. The employment of multidisciplinary teams to manage this subset of patients, address obstacles to self-care, diminish hospital readmissions, and enhance survival rates has been proposed. A multidisciplinary approach necessitates the comprehension of each team member’s roles and responsibilities, effective communication across diverse disciplines, and the utilization of appropriate shared decision-making processes. These are essential for establishing a diagnosis, monitoring patients for signs of improvement or exacerbation, prescribing medical interventions and lifestyle modifications, and educating patients and their caregivers.

## Figures and Tables

**Table 1:** RCTs on the use of mineralocorticoid receptor antagonists (MRAs)in HFpEF.

Trial	Design Study	Patients	Population Characteristics	LVEF	Groups of Treatment	Primary Endpoint (PE)	Results
**RANDOMIZED CLINICAL TRIALS**							
							Adj. Mean Diff; (95% CI)
Aldo-DHF [125]	Multicenter, randomized, placebo-controlled, double-blinded trial	422	HF NYHA II-III ambulatory	≥50%	Spironolactone 25 mg (*n* = 213) vs. placebo (*n* = 209)	Changes at 12 months FU E/e’ and VO2 peak	−1.5; −2.0–0.9; *p* < 0.001
							HR; (95% CI)
TOPCAT-trial [98]	Multicenter, international, randomized, double-blind, placebo-controlled trial	3445	HF NYHA II-III	≥45%		Composite of CV mortality, aborted cardiac arrest, or HHF	0.89; 0.77–1.04; *p* = 0.14
**ONGOING CLINICAL TRIALS**							
FINEARTS-HF (NCT04435626)	Multicenter, randomized, double-blind, parallel-group, placebo-controlled trial	6000	HF NYHA class II-IV Ambulatory or hospitalized On diuretics Structural heart abnormalities: ◦ LAD ≥ 3.8 cm ◦ LAA ≥ 20 cm^2^ ◦ LAVI > 30 mL/m^2^ ◦ LVMI ≥ 115 g/m^2^ (♂)/95 g/m^2^ (♀) ◦ Septal thickness or posterior wall thickness ≥ 1.1 cm NT-proBNP ≥ 300 pg/mL or BNP ≥ 100 pg/mL in sinus rhythm NT-proBNP ≥ 900 pg/mL or BNP ≥ 300 pg/mL in AF	≥40%	Finerenone vs placebo	Rate of CV death and HF events (HHF or urgent HF visit)	Ongoing
MIRACLE (NCT04595370)	Phase-2b, randomized, double-blind, active-controlled, multicenter study		HFNYHA II-III class CKD with eGFR 20–60 mL/min/1.73 m^2^	<60%	- Balcinrenone Dose A + dapagliflozin 10 mg - Balcinrenone Dose B + dapagliflozin 10 mg - Balcinrenone Dose C + dapagliflozin 10 mg - Dapagliflozin 10 m	Percent change from baseline in UACR at 12 weeks	Ongoing
SPIRIT-HF (NCT04727073)	Double-blind, randomized, placebo-controlled, parallel-group, interventional, phase-III study		Symptomatic HF NYHA II-IV class	Mid-range (40–49%) or preserved (≥50%)	Spironolactone (25–50 mg) vs. placebo	Composite of recurrent HHF and CV death	Ongoing
SPIRRIT-HFpEF (NCT02901184)	Registry-randomized clinical trial		Stable HF NYHA Class II-IV NT-proBNP > 300 ng/L (or BNP > 100 pg/mL) in sinus rhythm NT-proBNP > 750 ng/L (or BNP > 250 pg/mL) in AF NT-proBNP > 1200 ng/L (or BNP > 400 pg/mL) within the last 12 months, even if most recent value is lower. Regular use of LD	≥40%	Spironolactone vs. placebo	Primary outcome CV death or time to HHF	Ongoing

CV: cardiovascular; HHF heart failure hospitalizations; NYHA: New York Heart Association; HR: hazard ratio; CI: confidence interval; LAD: left atrial diameter; LAA: left atrial area; LAVI: left atrial volume index; LVMI: left ventricular mass index; NT-proBNP: n-terminal prohormone B-type natriuretic peptide; BNP: B-type natriuretic peptide; AF: atrial fibrillation; CKD: chronic kidney disease; UACR: urine albumin-to-creatinine ratio; and LD: loop diuretics.

## Supplementary Materials

The following supporting information can be downloaded at: https://www.mdpi.com/article/10.3390/jcm13051375/s1, Table S1. RCTs on the use of mineralocorticoid receptor antagonists (MRAs)in HFpEF [98,125]. Table S2. RCTs on SGLT2i use in HFpEF [51,52,133,134,137,138,139,140,141,142].
