# Supplementary material for: Heart Failure with Preserved Ejection Fraction: How to Deal with This Chameleon
Source: J Clin Med. 2024 Feb 28;13(5):1375. doi: 10.3390/jcm13051375 (PMC10933980; doi:10.3390/jcm13051375)
Supplement: Supplementary file 1 [file jcm-13-01375-s001.zip › jcm-2796478-supplementary.pdf]

Table S1. RCTs on the use of mineralocorticoid receptor antagonists (MRAs)in HFpEF.

Trial	Design Study	Patients	Population Characteristics	LVEF	Groups of Treatment	Primary Endpoint (PE)	Results
			<u>**RANDOMIZED CLINICAL TRIALS**</u>				
							Adj. Mean Diff; (95% CI)
Aldo-DHF [125]	Multicenter, randomized, placebo-controlled, double-blinded trial	422	• HF NYHA II-III ambulatory	≥50%	Spironolactone 25 mg (*n*= 213) vs. placebo (*n* = 209)	Changes at 12 months FU E/e′ andVO2 peak	−1.5; −2.0–0.9; *p*< 0.001
							HR; (95% CI)
TOPCAT-trial [98]	Multicenter, international, randomized, double-blind, placebo-controlled trial	3445	• HF NYHA II-III	≥45%		Composite ofCV mortality, aborted cardiac arrest, or HHF	0.89; 0.77–1.04; *p* = 0.14
			<u>**ONGOING CLINICAL TRIALS**</u>				
FINEARTS-HF (NCT04435626)	Multicenter, randomized, double-blind, parallel-group, placebo-controlled trial	6000	• HF NYHA class II-IV Ambulatory or hospitalized<br>• On diuretics<br>• Structural heart abnormalities:<br>○ LAD ≥3.8 cm<br>○ LAA ≥ 20 cm^2<br>○ LAVI >30 mL/m^2<br>○ LVMI ≥115 g/m^2 (♂)/95 g/m^2 (♀)<br>○ Septal thickness or posterior wall thickness ≥1.1 cm<br>• NT-proBNP ≥300 pg/mL or BNP ≥100 pg/mL in sinus rhythm<br>• NT-proBNP ≥900 pg/mL or BNP ≥300 pg/mL in AF	≥40%	Finerenone vs placebo	Rate of CV death and HF events (HHF or urgent HF visit)	Ongoing
MIRACLE (NCT04595370)	Phase-2b, randomized, double-blind, active-controlled, multicenterstudy		• HFNYHA II-III class<br>• CKD with eGFR 20–60 mL/min/1.73 m^2	<60%	- Balcinrenone Dose A + dapagliflozin 10 mg<br>- Balcinrenone Dose B + dapagliflozin 10 mg<br>- Balcinrenone Dose C + dapagliflozin 10 mg<br>- Dapagliflozin 10 m	Percent change from baseline in UACR at 12 weeks	Ongoing
SPIRIT-HF (NCT04727073)	Double-blind, randomized, placebo-controlled, parallel-group,interventio nal,phase-III study		• Symptomatic HF NYHA II-IV class	Mid-range (40–49%) or preserved (≥50%)	Spironolactone (25–50 mg) vs. placebo	Composite of recurrent HHF and CV death	Ongoing

SPIRRIT-HFpEF (NCT02901184)	Registry-randomized clinical trial	• Stable HF NYHA Class II-IV<br>• NT-proBNP >300 ng/L (or BNP >100 pg/mL) in sinus rhythm<br>• NT-proBNP >750 ng/L (or BNP >250 pg/mL) in AF<br>• NT-proBNP >1200 ng/L (or BNP >400 pg/mL) within the last 12 months, even if most recent value is lower.<br>• Regular use of LD	≥40%	Spironolactone vs. placebo	Primary outcome CV death or time to HHF	Ongoing

CV: cardiovascular; HHF heart failure hospitalizations; NYHA: New York Heart Association; HR: hazard ratio; CI: confidence interval; LAD: left atrial diameter; LAA: left atrial area; LAVI:left atrial volume index; LVMI: left ventricular mass index; NT-proBNP: n-terminal prohormone B-type natriuretic peptide; BNP: B-type natriuretic peptide;; AF: atrial fibrillation; CKD: chronic kidney disease; UACR: urine albumin-to-creatinine ratio; and LD: loop diuretics.

Table S2. RCTs on SGLT2i use in HFpEF.

	N° pt	DM	Follow-Up (Median)	Age	Sex (% Female)	LVEF*	Treatment	Primary Outcome	HR; (95% CI)
**EMPEROR-PRESERVED [51] NCT03057951**	5988	with or without DM	26.2 months	71.8	44.6%	54%	Empagliflozin	• CV deaths<br>• HHF	0.79; (0.69–0.90I) *p* <0.001
**PRESERVED-HF [133] NCT03030235**	324	with or without DM	3.0 years	70.0	57%	60%	Dapagliflozin	• KCCQ-CS at 12 weeks after treatment initiation	68.6; (66.2, 71.0) *p* 0.001
**DELIVER [52] NCT03619213.**	6263		2.3 year	71.8	43.6%	54%	Dapagliflozin	• Composite of worsening HF or CV death*	0.82; (0.73–0.92) *p* <0.001
**SOLOIST-WHF [134] NCT03521934.**	1222	with DM and recent HF worsening	9.0 months	70	32.6%	20% with LVEF >50%	Sotagliflozin	• Total number of CV deaths<br>• HHF<br>• HF urgent visits	0.67 (0.52–0.85); *p* <0.001
**EMPERIAL-PRESERVED NCT03448406 [137]**	315	with or without DM	12 weeks	73.5	43.2	53.1	Empagliflozin	• -6MWTD change in week 12	*p* =0.37
**CHIEF-HF NCT04252287 [138]**	476	with or without DM	2 weeks	63.4 ± 13.3	45%	50%(60%)	Canagliflozin (100 mg)	• Change in KCCQ TSS at 12 weeks	100 mg of canagliflozin or placebo
**VERTIS CV NCT01986881 [139]**	8246	with DM	3.5 years	64.4	30%	1007 patients with LVEF >45%)	Ertugliflozin	• CV death, nonfatal MI, nonfatal stroke	0.97 (0.85–1.11) *p*<0.001
**SCORED** [134].	10,584	with DM, CKD**	16 months	72	40%	>45%	Sotagliflozin	• risk of the composite of CV deaths, HHF	0.95 (0.86–1.05)
**DECLARED-TIMI-58 [140]**	17,160	with DM and CVD or multiple CVD risk factors	4.2 years	72	37%	1316 patients with 55%	dapagliflozin	• Primary safety outcome<br>○ Non-inferiority to placebo with respect to MACE	• Primary safety outcome: *p* <0.001 for noninferiorit y<br>• Primary efficacy outcomes:

								• Primary efficacy outcomes:<br>○ MACE: CV death, MI, or ischemic stroke<br>○ CV death or HHF	• **MACE** 0.93; 0.84–1.03; *p* =0.17<br>• **CV death or HHF** 0.83; 0.73–0.95; *p* =0.005)
**MUSCAT-HF NCT03315143 [141]**	160<br>• Luseogl ifloz(82)<br>• Voglibo se (83)	DM and HF	12 weeks			>45%	Luseogliflozin or voglibose	• Difference in change in BNP from baseline to 12 weeks between the patients receiving luseogliflozin and those receivingvogl ibose	0.93; 0.78–1.10; *p* =0.26
**CANDLE NCT03315143 [142]**	253	DM and stable HF	24 weeks	68	25%	≥50%(71%)	canagliflozin 100 mg or glimepiride	Percentage change (post/pre −1) from baseline in NT-proBNP at week 24.	0.48; −0.13–1.59, *p* = 0.226

LVEF: left ventricle ejection fraction; DM: diabetes mellitus; CHD: chronic kidney disease;HHF heart failurehospitalizations; MACE:major adverse cardiovascular events; BNP: brain natriuretic peptide; and KCCQ TSS:Kansas City Cardiomyopathy Questionnaire Total Symptom Score.
